# The genome sequence of the particolored bat, *Vespertilio murinus *Linnaeus, 1758

**DOI:** 10.12688/wellcomeopenres.22606.1

**Published:** 2024-07-26

**Authors:** Bob Vandendriessche, An Martel, Meike Mai, Emma C. Teeling, Sonja C. Vernes, Wellcome Sanger Institute Tree of Life Management, Samples and Laboratory team, Wellcome Sanger Institute Scientific Operations: Sequencing Operations, Wellcome Sanger Institute Tree of Life Core Informatics team, Tree of Life Core Informatics collective, Darwin Tree of Life Consortium

**Affiliations:** 1 Bat Working Group Natuurpunt Studie vzw, Mechelen, Belgium; 2 Wildlife Health Ghent, Faculty of Veterinary Medicine, Ghent University, Merelbeke, Belgium; 3 School of Biology, University of St Andrews, St Andrews, Scotland, UK; 4 University College Dublin, Dublin, Leinster, Ireland; 5 Wellcome Sanger Institute, Hinxton, England, UK

**Keywords:** Vespertilio murinus, particolored bat, genome sequence, chromosomal, Chiroptera

## Abstract

We present a genome assembly from an individual male *Vespertilio murinus* (the particolored bat; Chordata; Mammalia; Chiroptera; Vespertilionidae). The genome sequence is 1,925.6 megabases in span. Most of the assembly is scaffolded into 20 chromosomal pseudomolecules, including the X and Y sex chromosomes. The mitochondrial genome has also been assembled and is 16.96 kilobases in length.

## Species taxonomy

Eukaryota; Opisthokonta; Metazoa; Eumetazoa; Bilateria; Deuterostomia; Chordata; Craniata; Vertebrata; Gnathostomata; Teleostomi; Euteleostomi; Sarcopterygii; Dipnotetrapodomorpha; Tetrapoda; Amniota; Mammalia; Theria; Eutheria; Boreoeutheria; Laurasiatheria; Chiroptera; Yangochiroptera; Vespertilionidae; *Vespertilio*; *Vespertilio murinus* Linnaeus, 1758 (NCBI:txid59485).

## Background

**Figure 1.**
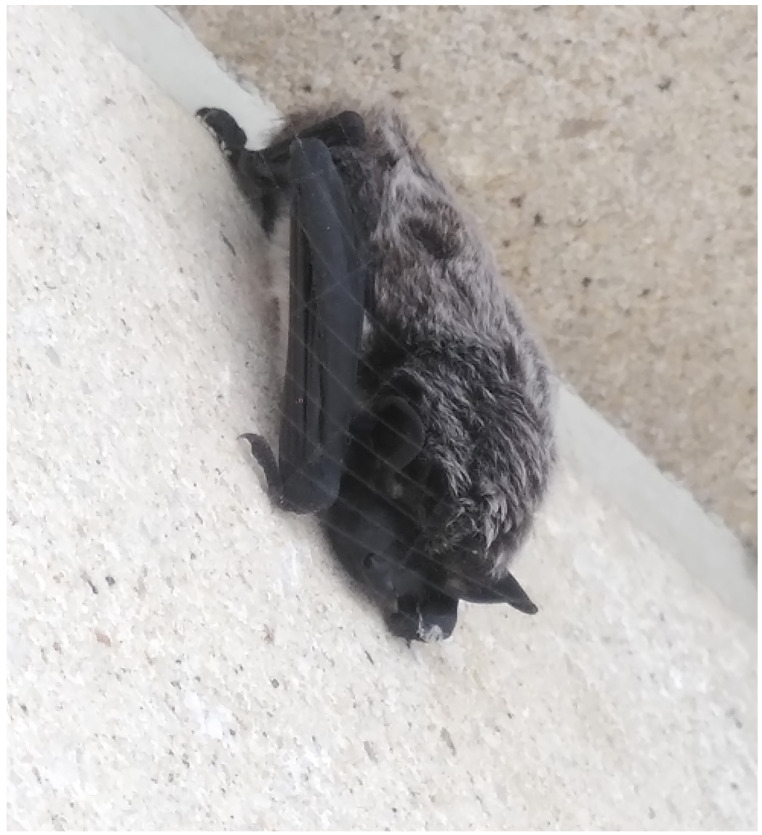
A male *Vespertilio murinus* resting on the outside of a large building at the seafront in Blankenberge, Belgium, 13 Sept. 2023. Along the North Sea coast, this behaviour has been observed frequently in this species - as it is in *Pipistrellus nathusii* - during migration. (Image © Bob Vandendriessche).

The Parti-coloured bat ( *Vespertilio murinus*) is a medium-sized bat, characterised by its long fur on the back that is unmistakably bicoloured: a dark brown base and silvery white tips ( Figure 1). Until the 20th century, the species has been confounded in literature with the Greater mouse-eared bat ( *Myotis myotis,* Borkhausen 1797) that originally was named *Vespertilio murinus* by Borkhausen, the reason for which some authors used the name ‘ *Vespertilio discolor’* for the Parti-coloured bat ( Rydell & Baagøe, 1994).

The species is listed by the IUCN Red list as “Least Concern” globally with a stable population trend ( Coroiu, 2016), although it is not considered common in large parts of its range. It has a wide distribution in the northern Palaearctic, from France and Britain in the west, through central, northern and eastern Europe and Siberia to the Pacific coast. The southern limit of its range passes through the Balkans, central Asia and China ( Coroiu, 2016). In parts of its range, it is known to migrate seasonally over long distances up to over 800 kilometres ( Masing, 1989) and single movements as far as 1787 kilometres are known ( Markovets *et al.*, 2004), and so are offshore detections ( Brabant *et al.*, 2020).

With a litter size of two, or rarely three, offspring that is typical for migratory species, the females have two pairs of functional teats, which is a unique feature of this species and distinguishes it from all other European bat species ( Safi, 2006). Overall, there is very little sexual dimorphism. The maximum life span has been reported to be 12 years, based on one ringed individual ( Červený & Bürger, 1989).

In summer, the species is known to roost mainly in buildings, exceptionally in trees. Remarkably, winter roosts are not known, apart from anecdotal findings of individuals ( Safi, 2006). Females prefer to forage over large open waters, while males also hunt along rivers, over large agricultural or urban areas. Its preference for open areas makes the species particularly vulnerable for wind turbines.

The genome of the particolored bat, *Vespertilio murinus*, was sequenced as part of the Darwin Tree of Life Project (DToL) project, the Bat1K Project and the Vertebrate Genomes Project (VGP). Here we present a chromosomally complete genome sequence for *Vespertilio murinus*, based on one male specimen from Ostend, Belgium.

## Genome sequence report

The genome was sequenced from a male *Vespertilio murinus* collected from Ostend, Belgium. A total of 44-fold coverage in Pacific Biosciences single-molecule HiFi long reads was generated. Primary assembly contigs were scaffolded with chromosome conformation Hi-C data. Manual assembly curation corrected 49 missing joins or mis-joins and removed 4 haplotypic duplications, reducing the scaffold number by 11.88%.

**Figure 2.**
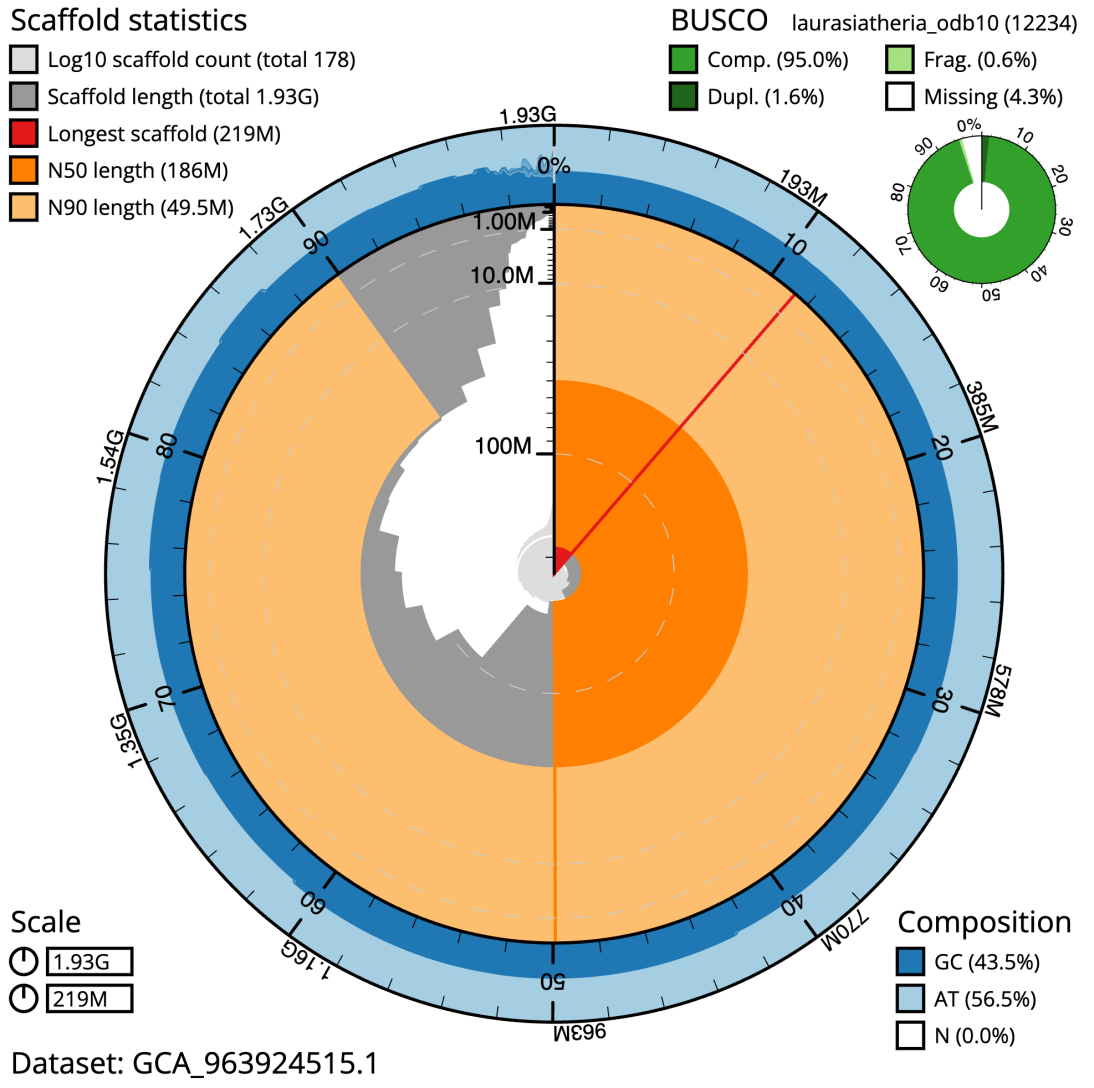
Genome assembly of *Vespertilio murinus*, mVesMur1.1: metrics. The BlobToolKit snail plot shows N50 metrics and BUSCO gene completeness. The main plot is divided into 1,000 size-ordered bins around the circumference with each bin representing 0.1% of the 1,925,577,803 bp assembly. The distribution of scaffold lengths is shown in dark grey with the plot radius scaled to the longest scaffold present in the assembly (219,009,580 bp, shown in red). Orange and pale-orange arcs show the N50 and N90 scaffold lengths (186,292,382 and 49,547,305 bp), respectively. The pale grey spiral shows the cumulative scaffold count on a log scale with white scale lines showing successive orders of magnitude. The blue and pale-blue area around the outside of the plot shows the distribution of GC, AT and N percentages in the same bins as the inner plot. A summary of complete, fragmented, duplicated and missing BUSCO genes in the laurasiatheria_odb10 set is shown in the top right. An interactive version of this figure is available at https://blobtoolkit.genomehubs.org/view/Vespertilio_murinus/dataset/GCA_963924515.1/snail.

**Figure 3.**
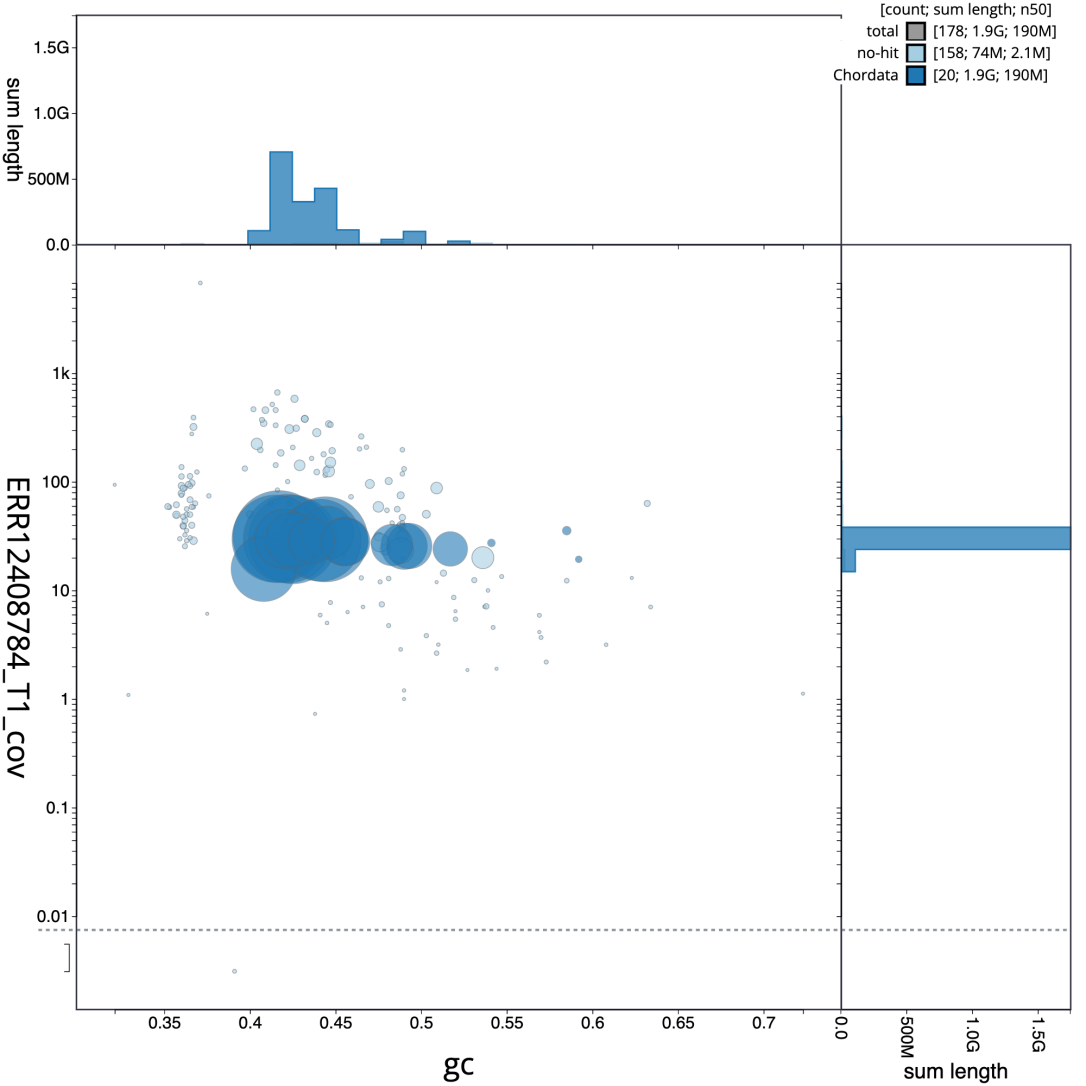
Genome assembly of *Vespertilio murinus*, mVesMur1.1: BlobToolKit GC-coverage plot. Sequences are coloured by phylum. Circles are sized in proportion to sequence length. Histograms show the distribution of sequence length sum along each axis. An interactive version of this figure is available at https://blobtoolkit.genomehubs.org/view/Vespertilio_murinus/dataset/GCA_963924515.1/blob.

**Figure 4.**
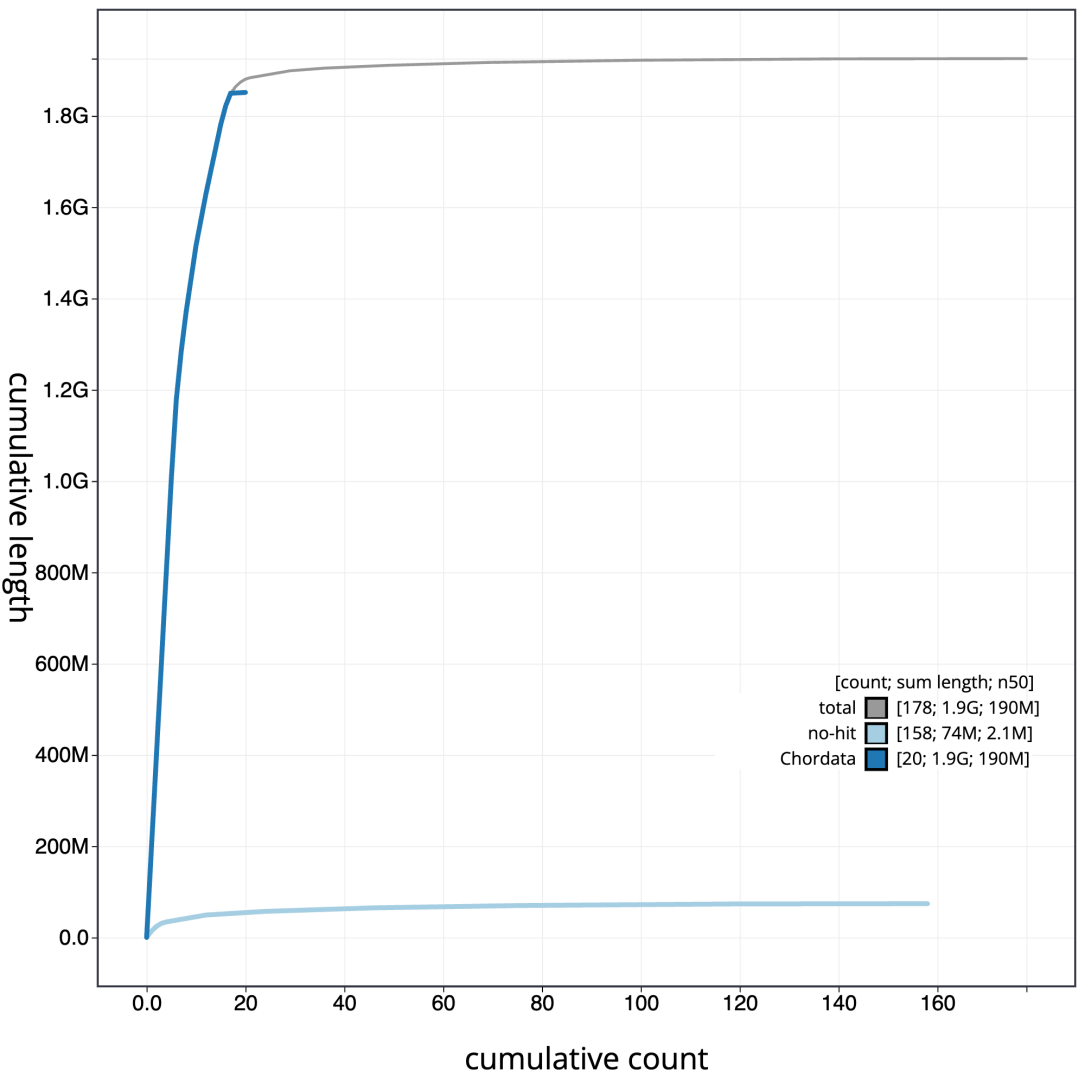
Genome assembly of *Vespertilio murinus* mVesMur1.1: BlobToolKit cumulative sequence plot. The grey line shows cumulative length for all sequences. Coloured lines show cumulative lengths of sequences assigned to each phylum using the buscogenes taxrule. An interactive version of this figure is available at https://blobtoolkit.genomehubs.org/view/Vespertilio_murinus/dataset/GCA_963924515.1/cumulative.

**Figure 5.**
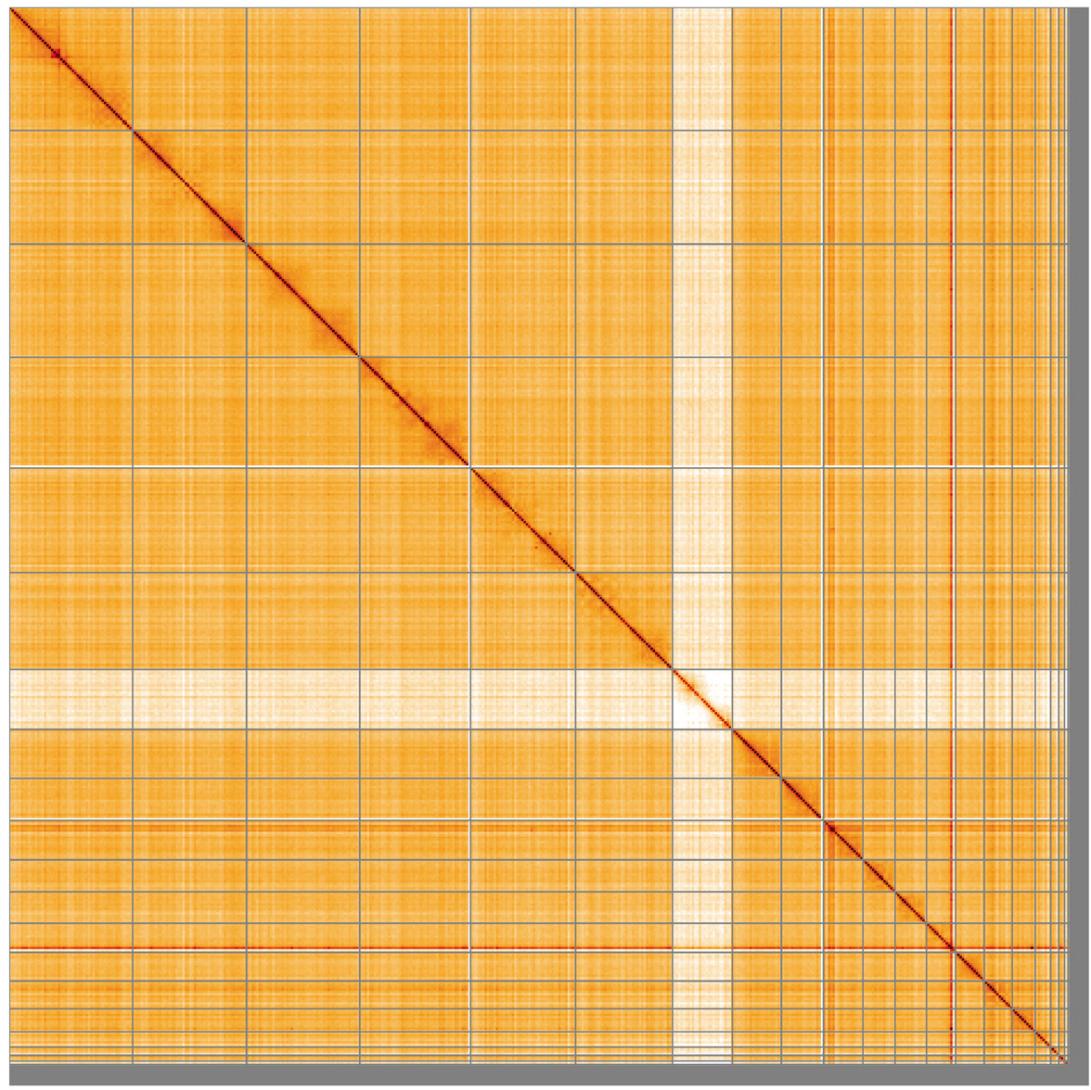
Genome assembly of *Vespertilio murinus* mVesMur1.1: Hi-C contact map of the mVesMur1.1 assembly, visualised using HiGlass. Chromosomes are shown in order of size from left to right and top to bottom. An interactive version of this figure may be viewed at https://genome-note-higlass.tol.sanger.ac.uk/l/?d=MQFBPCWcQBGnl_BQz6w8Fw.

The final assembly has a total length of 1,925.6 Mb in 177 sequence scaffolds with a scaffold N50 of 186.3 Mb ( Table 1). The snail plot in Figure 2 provides a summary of the assembly statistics, while the distribution of assembly scaffolds on GC proportion and coverage is shown in Figure 3. The cumulative assembly plot in Figure 4 shows curves for subsets of scaffolds assigned to different phyla. Most (97.66%) of the assembly sequence was assigned to 20 chromosomal-level scaffolds, representing 18 autosomes and the X and Y sex chromosomes. Chromosome-scale scaffolds confirmed by the Hi-C data are named in order of size ( Figure 5; Table 2). not fully phased, the assembly deposited is of one haplotype. Contigs corresponding to the second haplotype have also been deposited. The mitochondrial genome was also assembled and can be found as a contig within the multifasta file of the genome submission.

**Table 1.** Genome data for *Vespertilio murinus*, mVesMur1.1.

Project accession data		
Assembly identifier	mVesMur1.1	
Species	*Vespertilio murinus*	
Specimen	mVesMur1	
NCBI taxonomy ID	59485	
BioProject	PRJEB71534	
BioSample ID	Genome sequencing: SAMEA112247429 Hi-C scaffolding: SAMEA112247429	
Isolate information	mVesMur1	
Assembly metrics *		*Benchmark*
Consensus quality (QV)	61.8	*≥ 50*
*k*-mer completeness	100.0%	*≥ 95%*
BUSCO **	C:95.1%[S:93.5%,D:1.6%], F:0.7%,M:4.2%,n:12,234	*C ≥ 95%*
Percentage of assembly mapped to chromosomes	97.66%	*≥ 95%*
Sex chromosomes	XY	*localised homologous pairs*
Organelles	Mitochondrial genome: 16.96 kb	*complete single alleles*
Raw data accessions		
PacificBiosciences Revio	ERR12408784, ERR12408785	
Hi-C Illumina	ERR12512729	
PolyA RNA-Seq Illumina	ERR12512730	
Genome assembly		
Assembly accession	GCA_963924515.1	
*Accession of alternate haplotype*	GCA_963924695.1	
Span (Mb)	1,925.6	
Number of contigs	1,158	
Contig N50 length (Mb)	3.3	
Number of scaffolds	177	
Scaffold N50 length (Mb)	186.3	
Longest scaffold (Mb)	219.01	

* Assembly metric benchmarks are adapted from column VGP-2020 of “Table 1: Proposed standards and metrics for defining genome assembly quality” from Rhie *et al.* (2021). ** BUSCO scores based on the laurasiatheria_odb10 BUSCO set using version 5.4.3. C = complete [S = single copy, D = duplicated], F = fragmented, M = missing, n = number of orthologues in comparison. A full set of BUSCO scores is available at https://blobtoolkit.genomehubs.org/view/Vespertilio_murinus/dataset/GCA_963924515.1/busco.

**Table 2.** Chromosomal pseudomolecules in the genome assembly of *Vespertilio murinus*, mVesMur1.

INSDC accession	Name	Length (Mb)	GC%
OZ004704.1	1	219.01	41.5
OZ004705.1	2	202.11	42.0
OZ004706.1	3	201.28	42.5
OZ004707.1	4	196.85	41.5
OZ004708.1	5	186.29	44.5
OZ004709.1	6	172.14	44.0
OZ004711.1	7	86.93	42.0
OZ004712.1	8	74.91	42.5
OZ004713.1	9	70.16	44.5
OZ004714.1	10	56.84	45.5
OZ004715.1	11	55.96	45.5
OZ004716.1	12	51.82	49.0
OZ004717.1	13	50.86	43.5
OZ004718.1	14	49.55	49.5
OZ004719.1	15	40.76	48.5
OZ004720.1	16	27.46	51.5
OZ004721.1	17	14.38	49.0
OZ004722.1	18	10.12	53.5
OZ004710.1	X	106.65	41.0
OZ004723.1	Y	6.49	47.5
OZ004724.1	MT	0.02	37.0

The estimated Quality Value (QV) of the final assembly is 61.8 with *k*-mer completeness of 100.0%, and the assembly has a BUSCO v5.4.3 completeness of 95.1% (single = 93.5%, duplicated = %), using the laurasiatheria_odb10 reference set ( *n* = 12,234).

Metadata for specimens, barcode results, spectra estimates, sequencing runs, contaminants and pre-curation assembly statistics are given at https://links.tol.sanger.ac.uk/species/59485.

## Methods

### Sample acquisition and nucleic acid extraction

The specimen collected for this research, an adult male *Vespertilio murinus* (specimen ID SAN00002659, ToLID mVesMur1), was suffering upper arm (humerus) fracture, with a nearby wind turbine as a plausible cause. The animal was collected on 2022-09-08 by the Ostend Wildlife Rehab centre, who contacted the author (Bob Vandendriessche) for advice. It was transferred to the faculty of veterinary medicine (Ghent University) in Merelbeke, where it was euthanised following the AVMA Guidelines for the Euthanasia of Animals. After an inhalation anaesthetic overdose with isoflurane, sodium pentobarbital (0.11mg/g body weight) was administered intraperitoneally. Samples taken from the animal were preserved in RNA later at –70°C.

The workflow for high molecular weight (HMW) DNA extraction at the Wellcome Sanger Institute (WSI) Tree of Life Core Laboratory includes a sequence of core procedures: sample preparation; sample homogenisation, DNA extraction, fragmentation, and clean-up. In sample preparation, the mVesMur1 sample was weighed and dissected on dry ice ( Jay *et al.*, 2023). For sample homogenisation, tissue was cryogenically disrupted using the Covaris cryoPREP ^®^ Automated Dry Pulverizer ( Narváez-Gómez *et al.*, 2023).

HMW DNA was extracted using the Automated MagAttract v2 protocol ( Oatley *et al.*, 2023a). DNA was sheared into an average fragment size of 12–20 kb in a Megaruptor 3 system with speed setting 31 ( Bates *et al.*, 2023). Sheared DNA was purified by solid-phase reversible immobilisation ( Oatley *et al.*, 2023b): in brief, the method employs a 1.8X ratio of AMPure PB beads to sample to eliminate shorter fragments and concentrate the DNA. The concentration of the sheared and purified DNA was assessed using a Nanodrop spectrophotometer, Qubit Fluorometer and Qubit dsDNA High Sensitivity Assay kit. Fragment size distribution was evaluated by running the sample on the FemtoPulse system.

RNA was extracted from tissue of mVesMur1 in the Tree of Life Laboratory at the WSI using the RNA Extraction: Automated MagMax™ *mir*Vana protocol ( do Amaral *et al.*, 2023). The RNA concentration was assessed using a Nanodrop spectrophotometer and a Qubit Fluorometer using the Qubit RNA Broad-Range Assay kit. Analysis of the integrity of the RNA was done using the Agilent RNA 6000 Pico Kit and Eukaryotic Total RNA assay.

Protocols developed by the WSI Tree of Life laboratory are publicly available on protocols.io ( Denton *et al.*, 2023).

### Sequencing

Pacific Biosciences HiFi circular consensus DNA sequencing libraries were constructed according to the manufacturers’ instructions. Poly(A) RNA-Seq libraries were constructed using the NEB Ultra II RNA Library Prep kit. DNA and RNA sequencing was performed by the Scientific Operations core at the WSI on Pacific Biosciences Revio (HiFi) and Illumina NovaSeq 6000 (RNA-Seq) instruments. Hi-C data were also generated from muscle tissue of mVesMur1 using the Arima v2 kit. The Hi-C sequencing was performed using paired-end sequencing with a read length of 150 bp on the Illumina NovaSeq 6000 instrument.

### Genome assembly and curation

Assembly was carried out with Hifiasm ( Cheng *et al.*, 2021) and haplotypic duplication was identified and removed with purge_dups ( Guan *et al.*, 2020). The assembly was then scaffolded with Hi-C data ( Rao *et al.*, 2014) using YaHS ( Zhou *et al.*, 2023). The assembly was checked for contamination and corrected using the TreeVal pipeline ( Pointon *et al.*, 2023). Manual curation was performed using JBrowse2 ( Diesh *et al.*, 2023), HiGlass ( Kerpedjiev *et al.*, 2018) and PretextView ( Harry, 2022). The mitochondrial genome was assembled using MitoHiFi ( Uliano-Silva *et al.*, 2023), which runs MitoFinder ( Allio *et al.*, 2020) or MITOS ( Bernt *et al.*, 2013) and uses these annotations to select the final mitochondrial contig and to ensure the general quality of the sequence.

### Evaluation of final assembly

The final assembly was post-processed and evaluated with the three Nextflow ( Di Tommaso *et al.*, 2017) DSL2 pipelines “sanger-tol/readmapping” ( Surana *et al.*, 2023a), “sanger-tol/genomenote” ( Surana *et al.*, 2023b), and “sanger-tol/blobtoolkit” ( Muffato *et al.*, 2024). The pipeline sanger-tol/readmapping aligns the Hi-C reads with bwa-mem2 ( Vasimuddin *et al.*, 2019) and combines the alignment files with SAMtools ( Danecek *et al.*, 2021). The sanger-tol/genomenote pipeline transforms the Hi-C alignments into a contact map with BEDTools ( Quinlan & Hall, 2010) and the Cooler tool suite ( Abdennur & Mirny, 2020), which is then visualised with HiGlass ( Kerpedjiev *et al.*, 2018). It also provides statistics about the assembly with the NCBI datasets ( Sayers *et al.*, 2024) report, computes *k*-mer completeness and QV consensus quality values with FastK and MerquryFK, and a completeness assessment with BUSCO ( Manni *et al.*, 2021).

The sanger-tol/blobtoolkit pipeline is a Nextflow port of the previous Snakemake Blobtoolkit pipeline ( Challis *et al.*, 2020). It aligns the PacBio reads with SAMtools and minimap2 ( Li, 2018) and generates coverage tracks for regions of fixed size. In parallel, it queries the GoaT database ( Challis *et al.*, 2023) to identify all matching BUSCO lineages to run BUSCO ( Manni *et al.*, 2021). For the three domain-level BUSCO lineage, the pipeline aligns the BUSCO genes to the Uniprot Reference Proteomes database ( Bateman *et al.*, 2023) with DIAMOND ( Buchfink *et al.*, 2021) blastp. The genome is also split into chunks according to the density of the BUSCO genes from the closest taxonomically lineage, and each chunk is aligned to the Uniprot Reference Proteomes database with DIAMOND blastx. Genome sequences that have no hit are then chunked with seqtk and aligned to the NT database with blastn ( Altschul *et al.*, 1990). All those outputs are combined with the blobtools suite into a blobdir for visualisation.

All three pipelines were developed using the nf-core tooling ( Ewels *et al.*, 2020), use MultiQC ( Ewels *et al.*, 2016), and make extensive use of the Conda package manager, the Bioconda initiative ( Grüning *et al.*, 2018), the Biocontainers infrastructure ( da Veiga Leprevost *et al.*, 2017), and the Docker ( Merkel, 2014) and Singularity ( Kurtzer *et al.*, 2017) containerisation solutions.

Table 3 contains a list of relevant software tool versions and sources.

**Table 3.** Software tools: versions and sources.

Software tool	Version	Source
BEDTools	2.30.0	https://github.com/arq5x/bedtools2
Blast	2.14.0	ftp://ftp.ncbi.nlm.nih.gov/blast/executables/blast+/
BlobToolKit	4.3.7	https://github.com/blobtoolkit/blobtoolkit
BUSCO	5.4.3 and 5.5.0	https://gitlab.com/ezlab/busco
bwa-mem2	2.2.1	https://github.com/bwa-mem2/bwa-mem2
Cooler	0.8.11	https://github.com/open2c/cooler
DIAMOND	2.1.8	https://github.com/bbuchfink/diamond
fasta_windows	0.2.4	https://github.com/tolkit/fasta_windows
FastK	427104ea91c78c3b8b8b49f1a7d6bbeaa869ba1c	https://github.com/thegenemyers/FASTK
GoaT CLI	0.2.5	https://github.com/genomehubs/goat-cli
Hifiasm	0.16.1-r375	https://github.com/chhylp123/hifiasm
HiGlass	1.11.6	https://github.com/higlass/higlass
HiGlass	44086069ee7d4d3f6f3f0012569789ec138f42b84a a44357826c0b6753eb28de	https://github.com/higlass/higlass
MerquryFK	d00d98157618f4e8d1a9190026b19b471055b22e	https://github.com/thegenemyers/MERQURY.FK
MitoHiFi	2	https://github.com/marcelauliano/MitoHiFi
MultiQC	1.14, 1.17, and 1.18	https://github.com/MultiQC/MultiQC
NCBI Datasets	15.12.0	https://github.com/ncbi/datasets
Nextflow	23.04.0-5857	https://github.com/nextflow-io/nextflow
PretextView	0.2	https://github.com/wtsi-hpag/PretextView
purge_dups	1.2.3	https://github.com/dfguan/purge_dups
samtools	1.16.1, 1.17, and 1.18	https://github.com/samtools/samtools
sanger-tol/genomenote	1.1.1	https://github.com/sanger-tol/genomenote
sanger-tol/readmapping	1.2.1	https://github.com/sanger-tol/readmapping
Seqtk	1.3	https://github.com/lh3/seqtk
Singularity	3.9.0	https://github.com/sylabs/singularity
TreeVal	1.0.0	https://github.com/sanger-tol/treeval
YaHS	yahs-1.1.91eebc2	https://github.com/c-zhou/yahs

### Wellcome Sanger Institute – Legal and Governance

The materials that have contributed to this genome note have been supplied by a Darwin Tree of Life Partner. The submission of materials by a Darwin Tree of Life Partner is subject to the **‘Darwin Tree of Life Project Sampling Code of Practice’**, which can be found in full on the Darwin Tree of Life website here. By agreeing with and signing up to the Sampling Code of Practice, the Darwin Tree of Life Partner agrees they will meet the legal and ethical requirements and standards set out within this document in respect of all samples acquired for, and supplied to, the Darwin Tree of Life Project.

Further, the Wellcome Sanger Institute employs a process whereby due diligence is carried out proportionate to the nature of the materials themselves, and the circumstances under which they have been/are to be collected and provided for use. The purpose of this is to address and mitigate any potential legal and/or ethical implications of receipt and use of the materials as part of the research project, and to ensure that in doing so we align with best practice wherever possible. The overarching areas of consideration are:

• Ethical review of provenance and sourcing of the material

• Legality of collection, transfer and use (national and international)

Each transfer of samples is further undertaken according to a Research Collaboration Agreement or Material Transfer Agreement entered into by the Darwin Tree of Life Partner, Genome Research Limited (operating as the Wellcome Sanger Institute), and in some circumstances other Darwin Tree of Life collaborators.

## Data Availability

European Nucleotide Archive: Vespertilio murinus (particolored bat). Accession number PRJEB71534; https://identifiers.org/ena.embl/PRJEB71534 ( Wellcome Sanger Institute, 2024). The genome sequence is released openly for reuse. The *Vespertilio murinus* genome sequencing genome sequencing initiative is part of the Darwin Tree of Life (DToL) project, the Bat1K Project and the Vertebrate Genomes Project (VGP). All raw sequence data and the assembly have been deposited in INSDC databases. The genome will be annotated using available RNA-Seq data and presented through the Ensembl pipeline at the European Bioinformatics Institute. Raw data and assembly accession identifiers are reported in Table 1.

## Author information

Members of the Darwin Tree of Life Barcoding collective are listed here: https://doi.org/10.5281/zenodo.4893703.

Members of the Wellcome Sanger Institute Tree of Life Management, Samples and Laboratory team are listed here: https://doi.org/10.5281/zenodo.10066175.

Members of Wellcome Sanger Institute Scientific Operations: Sequencing Operations are listed here: https://doi.org/10.5281/zenodo.10043364.

Members of the Wellcome Sanger Institute Tree of Life Core Informatics team are listed here: https://doi.org/10.5281/zenodo.10066637.

Members of the Tree of Life Core Informatics collective are listed here: https://doi.org/10.5281/zenodo.5013541.

Members of the Darwin Tree of Life Consortium are listed here: https://doi.org/10.5281/zenodo.4783558.

## References

[ref-1] Abdennur N Mirny LA : Cooler: scalable storage for Hi-C data and other genomically labeled arrays. *Bioinformatics.* 2020;36(1):311–316. 10.1093/bioinformatics/btz540 31290943 PMC8205516

[ref-2] Allio R Schomaker-Bastos A Romiguier J : MitoFinder: efficient automated large-scale extraction of mitogenomic data in target enrichment phylogenomics. *Mol Ecol Resour.* 2020;20(4):892–905. 10.1111/1755-0998.13160 32243090 PMC7497042

[ref-3] Altschul SF Gish W Miller W : Basic local alignment search tool. *J Mol Biol.* 1990;215(3):403–410. 10.1016/S0022-2836(05)80360-2 2231712

[ref-4] Bateman A Martin MJ Orchard S : UniProt: the universal protein knowledgebase in 2023. *Nucleic Acids Res.* 2023;51(D1):D523–D531. 10.1093/nar/gkac1052 36408920 PMC9825514

[ref-5] Bates A Clayton-Lucey I Howard C : Sanger Tree of Life HMW DNA fragmentation: diagenode Megaruptor ^®^3 for LI PacBio. *protocols.io.* 2023. 10.17504/protocols.io.81wgbxzq3lpk/v1

[ref-6] Bernt M Donath A Jühling F : MITOS: improved *de novo* metazoan mitochondrial genome annotation. *Mol Phylogenet Evol.* 2013;69(2):313–319. 10.1016/j.ympev.2012.08.023 22982435

[ref-7] Brabant R Laurent Y Lafontaine RM : First offshore observation of parti-coloured bat *Vespertilio murinus* in the Belgian part of the North Sea. *Belg J Zool.* 2020;146(1):62. 10.26496/bjz.2016.40

[ref-8] Buchfink B Reuter K Drost HG : Sensitive protein alignments at Tree-of-Life scale using DIAMOND. *Nat Methods.* 2021;18(4):366–368. 10.1038/s41592-021-01101-x 33828273 PMC8026399

[ref-9] Červený J Bürger P : Density and structure of the bat community occupying an old parkat Žihobce (Czechoslovakia).In: Hanák, V., Horácek, I., and Gaisler, J. (eds.) *European bat research 1987*. Prague: Charles University Press,1989;475–486.

[ref-10] Challis R Kumar S Sotero-Caio C : Genomes on a Tree (GoaT): a versatile, scalable search engine for genomic and sequencing project metadata across the eukaryotic Tree of Life [version 1; peer review: 2 approved]. *Wellcome Open Res.* 2023;8:24. 10.12688/wellcomeopenres.18658.1 36864925 PMC9971660

[ref-11] Challis R Richards E Rajan J : BlobToolKit – interactive quality assessment of genome assemblies. *G3 (Bethesda).* 2020;10(4):1361–1374. 10.1534/g3.119.400908 32071071 PMC7144090

[ref-12] Cheng H Concepcion GT Feng X : Haplotype-resolved *de novo* assembly using phased assembly graphs with hifiasm. *Nat Methods.* 2021;18(2):170–175. 10.1038/s41592-020-01056-5 33526886 PMC7961889

[ref-13] Coroiu I : *Vespertilio murinus*, the IUCN Red List of threatened species 2016: e.T22947A22071456. 2016; [Accessed 4 June 2024]. 10.2305/IUCN.UK.2016-2.RLTS.T22947A22071456.en

[ref-14] da Veiga Leprevost F Grüning BA Alves Aflitos S : BioContainers: an open-source and community-driven framework for software standardization. *Bioinformatics.* 2017;33(16):2580–2582. 10.1093/bioinformatics/btx192 28379341 PMC5870671

[ref-15] Danecek P Bonfield JK Liddle J : Twelve years of SAMtools and BCFtools. *GigaScience.* 2021;10(2): giab008. 10.1093/gigascience/giab008 33590861 PMC7931819

[ref-16] Denton A Yatsenko H Jay J : Sanger Tree of Life wet laboratory protocol collection V.1. *protocols.io.* 2023. 10.17504/protocols.io.8epv5xxy6g1b/v1

[ref-17] Di Tommaso P Chatzou M Floden EW : Nextflow enables reproducible computational workflows. *Nat Biotechnol.* 2017;35(4):316–319. 10.1038/nbt.3820 28398311

[ref-18] Diesh C Stevens GJ Xie P : JBrowse 2: a modular genome browser with views of synteny and structural variation. *Genome Biol.* 2023;24(1): 74. 10.1186/s13059-023-02914-z 37069644 PMC10108523

[ref-19] do Amaral RJV Bates A Denton A : Sanger Tree of Life RNA extraction: automated MagMax™ mirVana. *protocols.io.* 2023. 10.17504/protocols.io.6qpvr36n3vmk/v1

[ref-20] Ewels P Magnusson M Lundin S : MultiQC: summarize analysis results for multiple tools and samples in a single report. *Bioinformatics.* 2016;32(19):3047–3048. 10.1093/bioinformatics/btw354 27312411 PMC5039924

[ref-21] Ewels PA Peltzer A Fillinger S : The nf-core framework for community-curated bioinformatics pipelines. *Nat Biotechnol.* 2020;38(3):276–278. 10.1038/s41587-020-0439-x 32055031

[ref-22] Grüning B Dale R Sjödin A : Bioconda: sustainable and comprehensive software distribution for the life sciences. *Nat Methods.* 2018;15(7):475–476. 10.1038/s41592-018-0046-7 29967506 PMC11070151

[ref-23] Guan D McCarthy SA Wood J : Identifying and removing haplotypic duplication in primary genome assemblies. *Bioinformatics.* 2020;36(9):2896–2898. 10.1093/bioinformatics/btaa025 31971576 PMC7203741

[ref-24] Harry E : PretextView (Paired Read Texture Viewer): a desktop application for viewing pretext contact maps. 2022; [Accessed 19 October 2022]. Reference Source

[ref-25] Jay J Yatsenko H Narváez-Gómez JP : Sanger Tree of Life sample preparation: triage and dissection. *protocols.io.* 2023. 10.17504/protocols.io.x54v9prmqg3e/v1

[ref-26] Kerpedjiev P Abdennur N Lekschas F : HiGlass: web-based visual exploration and analysis of genome interaction maps. *Genome Biol.* 2018;19(1): 125. 10.1186/s13059-018-1486-1 30143029 PMC6109259

[ref-27] Kurtzer GM Sochat V Bauer MW : Singularity: scientific containers for mobility of compute. *PLoS One.* 2017;12(5): e0177459. 10.1371/journal.pone.0177459 28494014 PMC5426675

[ref-28] Li H : Minimap2: pairwise alignment for nucleotide sequences. *Bioinformatics.* 2018;34(18):3094–3100. 10.1093/bioinformatics/bty191 29750242 PMC6137996

[ref-29] Manni M Berkeley MR Seppey M : BUSCO update: novel and streamlined workflows along with broader and deeper phylogenetic coverage for scoring of eukaryotic, prokaryotic, and viral genomes. *Mol Biol Evol.* 2021;38(10):4647–4654. 10.1093/molbev/msab199 34320186 PMC8476166

[ref-30] Markovets M Zelenova NP Shapoval AP : Beringung von Fledermäusen in der biologischen station Rybachy, 1957 – 2001. *Nyctalus (N.F.).* 2004;9(3):259–268. Reference Source

[ref-31] Masing M : A long distance flight of *Vespertilio murinus* from Estonia. *Myotis.* 1989;27:147–150. Reference Source

[ref-32] Merkel D : Docker: lightweight Linux containers for consistent development and deployment. *Linux J.* 2014;2014(239): 2. Reference Source

[ref-33] Muffato M Butt Z Challis R : sanger-tol/blobtoolkit: v0.3.0 – Poliwag. 2024. 10.5281/zenodo.10649272

[ref-34] Narváez-Gómez JP Mbye H Oatley G : Sanger Tree of Life sample homogenisation: covaris cryoPREP ^®^ automated dry pulverizer V.1. *protocols.io.* 2023. 10.17504/protocols.io.eq2lyjp5qlx9/v1

[ref-35] Oatley G Denton A Howard C : Sanger Tree of Life HMW DNA extraction: automated MagAttract v.2. *protocols.io.* 2023a. 10.17504/protocols.io.kxygx3y4dg8j/v1

[ref-36] Oatley G Sampaio F Howard C : Sanger Tree of Life fragmented DNA clean up: automated SPRI. *protocols.io.* 2023b; [Accessed 21 November 2023]. 10.17504/protocols.io.q26g7p1wkgwz/v1

[ref-37] Pointon DL Eagles W Sims Y : sanger-tol/treeval v1.0.0 – Ancient Atlantis. 2023. 10.5281/zenodo.10047654

[ref-38] Quinlan AR Hall IM : BEDTools: a flexible suite of utilities for comparing genomic features. *Bioinformatics.* 2010;26(6):841–842. 10.1093/bioinformatics/btq033 20110278 PMC2832824

[ref-39] Rao SSP Huntley MH Durand NC : A 3D map of the human genome at kilobase resolution reveals principles of chromatin looping. *Cell.* 2014;159(7):1665–1680. 10.1016/j.cell.2014.11.021 25497547 PMC5635824

[ref-40] Rhie A McCarthy SA Fedrigo O : Towards complete and error-free genome assemblies of all vertebrate species. *Nature.* 2021;592(7856):737–746. 10.1038/s41586-021-03451-0 33911273 PMC8081667

[ref-41] Rydell J Baagøe HJ : Vespertilio murinus. *Mammalian Species.* 1994;4(67):1–6.

[ref-42] Safi K : Die Zweifarbfledermaus in der Schweiz: status und Grundlagen zum Schutz. Bern: Haupt Verlag,2006. Reference Source

[ref-43] Sayers EW Cavanaugh M Clark K : GenBank 2024 update. *Nucleic Acids Res.* 2024;52(D1):D134–D137. 10.1093/nar/gkad903 37889039 PMC10767886

[ref-44] Surana P Muffato M Qi G : sanger-tol/readmapping: sanger-tol/readmapping v1.1.0 - Hebridean Black (1.1.0). *Zenodo.* 2023a. 10.5281/zenodo.7755669

[ref-45] Surana P Muffato M Sadasivan Baby C : sanger-tol/genomenote (v1.0.dev). *Zenodo.* 2023b. 10.5281/zenodo.6785935

[ref-46] Uliano-Silva M Ferreira JGRN Krasheninnikova K : MitoHiFi: a python pipeline for mitochondrial genome assembly from PacBio high fidelity reads. *BMC Bioinformatics.* 2023;24(1): 288. 10.1186/s12859-023-05385-y 37464285 PMC10354987

[ref-47] Vasimuddin M Misra S Li H : Efficient architecture-aware acceleration of BWA-MEM for multicore systems. In: *2019 IEEE International Parallel and Distributed Processing Symposium (IPDPS)*. IEEE,2019;314–324. 10.1109/IPDPS.2019.00041

[ref-48] Wellcome Sanger Institute: The genome sequence of the particolored bat, *Vespertilio murinus* Linnaeus, 1758. European Nucleotide Archive. [dataset], accession number PRJEB71534,2024. 10.12688/wellcomeopenres.22606.1 PMC11375412 39239168

[ref-49] Zhou C McCarthy SA Durbin R : YaHS: Yet another Hi-C Scaffolding tool. *Bioinformatics.* 2023;39(1): btac808. 10.1093/bioinformatics/btac808 36525368 PMC9848053

