# Supplementary material for: The genome sequence of the particolored bat, Vespertilio murinus Linnaeus, 1758
Source: Wellcome Open Res. 2024 Jul 26;9:403. [Version 1] doi: 10.12688/wellcomeopenres.22606.1 (PMC11375412; doi:10.12688/wellcomeopenres.22606.1)
Supplement: Peer review history

# Reviewer report: Reviewer response for version 1

**DOI:** 10.21956/wellcomeopenres.24905.r95350

**Published:** 2024-11-21

**Authors:** Xiuguang Mao

**Affiliations:** 1 School of Ecological and Environmental Sciences, East China Normal University, Shanghai, China

This manuscript presents a high-quality chromosome-scale assembly for *Vespertilio murinus* and this genomic resource can be very useful for the study on genetic basis of migration in this species. The total number of assembled chromosomal-scale scaffolds corresponds to the haploid chromosome number of this species (2n=38).

- I suggest authors to cite one previous cytogenetic study on this species (e.g. Kulemzina et al. 2011. Cytogenetic and Genome Research, 134(3), 200-205).
- In addition, “not fully phased, the assembly deposited is of one haplotype.” Missing “While” in the front of this sentence?

Are sufficient details of methods and materials provided to allow replication by others?

Yes

Is the rationale for creating the dataset(s) clearly described?

Yes

Are the datasets clearly presented in a useable and accessible format?

Yes

Are the protocols appropriate and is the work technically sound?

Yes

Reviewer Expertise:

Comparative genomics, genome assembly, evolutionary biology, speciation, bats

I confirm that I have read this submission and believe that I have an appropriate level of expertise to confirm that it is of an acceptable scientific standard.

## References

[1] : Comparative chromosome painting of four Siberian Vespertilionidae species with Aselliscus stoliczkanus and human probes. *Cytogenet Genome Res* .2011;134(3) : 10.1159/000328834 200-5 10.1159/000328834 21709413

# Reviewer report: Reviewer response for version 1

**DOI:** 10.21956/wellcomeopenres.24905.r95352

**Published:** 2024-09-04

**Authors:** Wenhua Yu

**Affiliations:** 1 Guangzhou University, Guangzhou, China

This manuscript reports the genomic information of *Vespertilio murinus*, which can provide important foundational data for subsequent comparative genomics and genomic analyses. The analysis methods used in the paper are reasonable, the structure is complete, and the data is detailed. I have a few minor suggestions for the authors to consider and revise in order to further enhance the quality of the paper.

1. In Figure 1, is the grids a spider web? It is suggested to clarify this in the figure legend because it is odd. 
2. It is recommended to supplement an explanation of the tissue from which the genome was derived.
3. Please check the data in the figures, tables, and text for consistency; there is a discrepancy in the number of scaffolds.
4. I personally suggest that the authors supplement the annotation work, as this is crucial for future research.

Are sufficient details of methods and materials provided to allow replication by others?

Yes

Is the rationale for creating the dataset(s) clearly described?

Yes

Are the datasets clearly presented in a useable and accessible format?

Yes

Are the protocols appropriate and is the work technically sound?

Yes

Reviewer Expertise:

Comparative genomics, Phylogeny, Taxonomy, Evolutionary biology, Diversification, Bats

I confirm that I have read this submission and believe that I have an appropriate level of expertise to confirm that it is of an acceptable scientific standard.

# Reviewer report: Reviewer response for version 1

**DOI:** 10.21956/wellcomeopenres.24905.r95357

**Published:** 2024-09-02

**Authors:** Jiang Feng

**Affiliations:** 1 Northeast Normal University, Changchun, China

The authors in this manuscript describe a new reference genome of the particolored bat by using the HiFi circular consensus sequencing and Hi-C technologies. The genome has not been previously published to this reviewer’s knowledge and may be an important resource for Chiroptera and future comparative studies. The methods are appropriate and the genome sequence report is concise. In general, the study would be of interest to those who are working on bats and biodiversity.

**Some concerns and suggestions are listed as follows:**

 1. Latin name of the particolored bat is *Vespertilio murinus* in this manuscript, however, the Latin name of *Vespertilio sinensis* was also used in previously reported study. These two Latin names corresponding to the same common name, the particolored bat, do you think they are the same species? if yes, which Latin name is more accurate?

 2. Authors wrote that “Samples taken from the animal were preserved in RNA later at –70°C”, which samples of this bat were used? Added this information in the method.

 3. The methodology used here is nevertheless not completely clear, details should be provided in their method. For example, it is difficult for others to repeat their k-mer and BUSCO analyses. Versions of software and detailed parameters should be added in the method part.

 4. For the diploid organisms, did you meet the influence caused by the homologous sequences during assembly and Hi-C analysis? If yes, how to deal with?

 5. There are 177 scaffolds as described in the main text, however, 178 scaffold in Figure 2, please check.

 6. The chromosome id should be provided in Figure 5.

 7. Why no annotation information was contained in this study? I think a high-quality annotated genome will be a valuable resource for following genomic analyses across species, for population genomics, and for future evolutionary investigations, which could improve the significance of genome sequencing.

Are sufficient details of methods and materials provided to allow replication by others?

Partly

Is the rationale for creating the dataset(s) clearly described?

Yes

Are the datasets clearly presented in a useable and accessible format?

Yes

Are the protocols appropriate and is the work technically sound?

Yes

Reviewer Expertise:

animal behavior; animal ecology

I confirm that I have read this submission and believe that I have an appropriate level of expertise to confirm that it is of an acceptable scientific standard.
